# Topography and Morphology of Gastric Cancer in Nigeria: A Dual Institution Review of 622 Upper Gastrointestinal Endoscopies

**DOI:** 10.7759/cureus.14693

**Published:** 2021-04-26

**Authors:** Emeka Ray-Offor, Christopher C Obiorah

**Affiliations:** 1 Digestive Disease Unit, Oak Endoscopy Centre, Port Harcourt, NGA; 2 Department of Surgery, University of Port Harcourt Teaching Hospital, Port Harcourt, NGA; 3 Department of Anatomical Pathology, University of Port Harcourt Teaching Hospital, Port Harcourt, NGA

**Keywords:** gastric cancer, topography, morphology

## Abstract

Introduction

Gastric cancer is a leading cause of cancer mortality worldwide. The burden of this disease is highest in developing countries of East Asia, Eastern Europe, and parts of Central and Southern America. Africa, despite having a similar high profile of Helicobacter pylori infection with East Asia, has a reported low prevalence of gastric cancer. There is a paucity of data on the natural history and endoscopic presentation of gastric cancer in West Africa.

Aim

To study the topography and morphology of gastric cancer from two institutions in Southern Nigeria.

Methods

A cross-sectional retrospective study of 622 consecutive cases of upper gastrointestinal (GI) endoscopy performed in two referral endoscopy facilities in Port Harcourt, Rivers State, Nigeria from February 2012 to January 2021. Variables collated from centre records included age, sex, ethnicity, symptoms, site, endoscopic classification, and histology of gastric cancers. Statistical analysis was performed using IBM SPSS version 20 (IBM Corp., Armonk, NY).

Results

There were 17 (2.7%) cases of histologically confirmed gastric cancer. The age range of patients was from 34 years to 99 years (mean 60.7 ± 14.6 years). There were nine males and eight females (M:F ratio of 1.1:1). Antrum and cardia were predominantly affected in 10 (60.0%) and seven (6.7%) cases, respectively. Borrmann type 1 advanced gastric tumor was seen in seven (53.8%) and adenocarcinoma, the predominant histology, in 14 (82.4%) cases. Helicobacter pylori was detected in a sole case of gastric cancer.

Conclusion

Gastric cancer is uncommon in our environment and with a delayed presentation. A predominance of gastric antrum topography and exophytic growth morphology is the pattern.

## Introduction

Worldwide, gastric cancer is estimated as the third leading cause of cancer mortality in males and fourth in females accounting for 8% of all new cancer cases in 2008 [1]. The burden of this disease is highest in developing countries of East Asia, Eastern Europe, and parts of Central and Southern America, but low incidence of gastric cancer is reported in North America and most parts of Africa [1]. Screening policy in endemic regions and favorable trends in chronic Helicobacter pylori infection reduction have proven to be effective in improving prognosis of this disease [2-4]. Epidemiological reports from different regions of Nigeria are varied with gastric cancer accounting for 7.8% to 18.4% of gastrointestinal cancers [5-7]. Meanwhile, the clinical presentation is generally of advanced disease often complicated by gastric outlet obstruction, haematemesis or perforation [8,9]. An early detection is essential as the prognosis of early gastric cancer is good with a 5-year survival rate of over 90% [10].

The risk factors for gastric cancer include age >40 years; Helicobacter pylori infection; previous precancerous diseases like chronic atrophic gastritis, gastric polyps, gastric ulcer, and pernicious anaemia; dietary and social habits such as alcohol consumption, smoking, high salt, preserved food intake [11]. Helicobacter pylori infection is implicated in the premalignant transformation through chronic atrophic gastritis, intestinal metaplasia to dysplastic change. While H. pylori affects over 50% of humans in developed countries, its prevalence reaches 80% or more among adults in Africa yet a low incidence of gastric cancer is reported [12]. The advent of next-generation sequencing and other genomic technologies have revealed gastric cancer as characterized by epidemiologic and histologic differences across countries [13]. Gene mutations, chromosomal aberrations, differential gene expression and epigenetic alterations including DNA/histone methylation are some of the genetic/epigenetic inﬂuences on gastric cancer pathogenesis [14].

Over the last decades, oesophagogastroduodenoscopy (OGD) has become the initial investigation of choice in patients with upper gastro-intestinal symptoms [15]. The detection of an abnormal mucosa in upper gastrointestinal endoscopy refers to the obvious elevation or depression, mucosal discoloration, or interruption in the course of superficial capillaries [16]. However, non-polypoid lesions might be missed when the endoscopists lack cognitive knowledge and training. White light endoscopy is associated with a significant miss rate for non-polypoid lesions. Image enhancing endoscopy technology includes magnification endoscopy, chromoendoscopy, narrow band imaging, auto-fluorescence and confocal microscopy [17,18].

There is a paucity of data on the natural history and endoscopic presentation of gastric cancer in West Africa. This study aims to study the topography and morphology of gastric cancers in two institutions of Southern Nigeria.

## Materials and methods

Study design and setting

This was a cross sectional, retrospective, and observational study conducted in Port Harcourt, Nigeria. Port Harcourt is the fifth largest city in Nigeria and the capital of Rivers State lying along the Bonny River which is in the Niger Delta area with a population of 1,148,665 [19]. Data was collated for all consecutive patients that underwent upper gastrointestinal endoscopy at two referral centres from a Surgical endoscopy service spanning from February 2012 to January 2021. An ethical approval was obtained from the Ethics Committee of the study centre. The exclusion criterion was paediatric patients with age below 18 years. The variables collated from the centres’ records were demographics, symptoms, site, morphologic classification, and histology of gastric cancer cases.

Endoscopy equipment

This comprised Karl Storz (Germany) video gastroscopes 13801/13821 PKS, Camera unit, 100W Xenon light source/pump, HD monitor and AIDA data capture device.

Procedure

An informed consent was obtained from each patient according to Helsinki declaration. A sedation/analgesia protocol of intravenous benzodiazepine (diazepam 2.5mg-10mg) and pharyngeal local anaesthesia using 10% lignocaine local spray was followed. Non-cooperative patients had general anaesthesia administered under the supervision of an anaesthesiologist. A systematic examination of the stomach on intubating the gastroesophageal junction was performed, then an intubation of the duodenum was performed. A retroflexion of the endoscope on withdrawal of endoscope from the duodenum into the stomach was done and a careful inspection of the cardia and fundus of the stomach was made. A video recording of each procedure was done and relevant image captured for documentation of findings.

Histology

A minimum of four biopsies were taken: two from antrum and two from body and targeted mucosal lesions. The biopsy tissue was immediately fixed in 10% buffered neutral formalin and transported to the histopathology laboratory. The specimen was processed by embedment in paraffin wax, microtome-sliced, then stained with haematoxylin and eosin stain. An examination by the pathologist (CCO) was performed under the microscope.

Statistical analysis

Statistical analysis was performed using IBM SPSS version 20 (IBM Corp., Armonk, NY). The mean age and standard deviation were calculated. Numerical and categorical data were represented in simple percentages.

## Results

A total of 622 OGDs were included in the study. There were multiple oesophageal, gastric and duodenal pathologies recorded with 17 (2.7%) gastric cancer cases.

**Figure 1:**
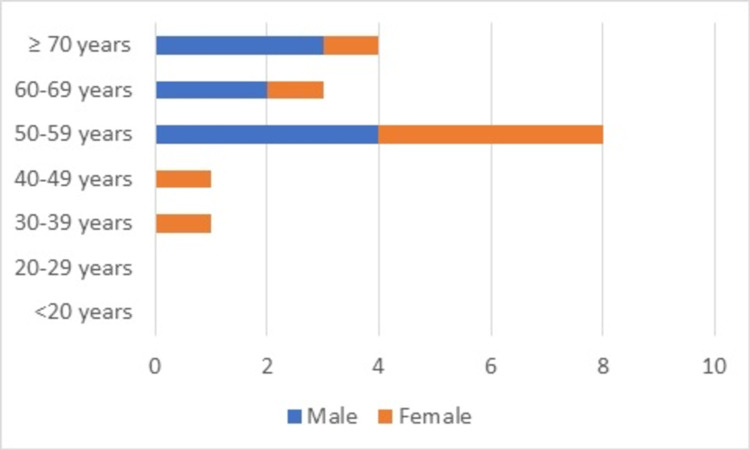
Age and sex distribution of gastric cancer cases

The age range of gastric cancer patients was from 34 years to 99 years. The mean age was 60.7 ± 14.6 years, median 59 years, and mode 52 years. There were nine males and eight females (M:F ratio of 1.1:1). This demographic is shown in Figure 1.

The ethnicity of the gastric cancer patients broadly reflected the tribes in Southern Nigeria: six Igbos; two Kalabaris; two Ikwerres; and two Ibibios. A sole case each was recorded for the following tribes: Etch; Anioma; Annang; Isoko; and Yoruba.

**Figure 2:**
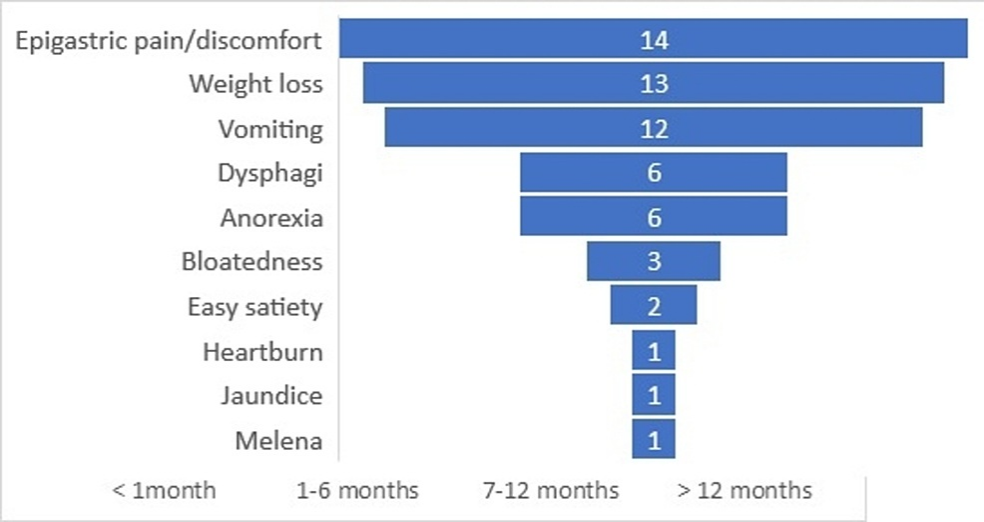
Symptoms in gastric cancer cases

The duration of presenting complaint(s) was from one month to six months in 10 cases and seven months to 12 months in seven cases. There was no asymptomatic case and no duration of symptom under one month was recorded among the patients with gastric cancer. A total of 59 complaints were recorded among 17 patients with gastric cancer. The leading complaints were epigastric pain/discomfort 14/59 (23.7%), weight loss 13/59 (22.0%) and vomiting 12/59 (20.3%) (Figure 2).

**Figure 3:**
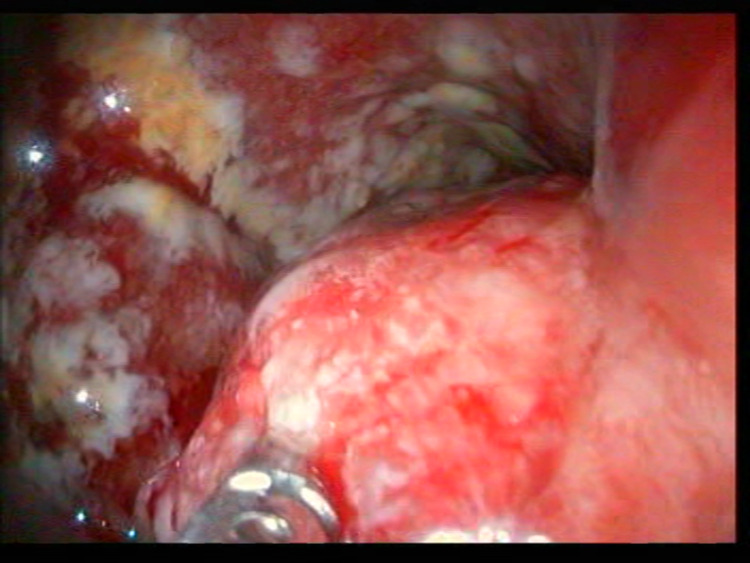
Endoscopic biopsy of Borrmann Type I antral gastric tumour

**Figure 4:**
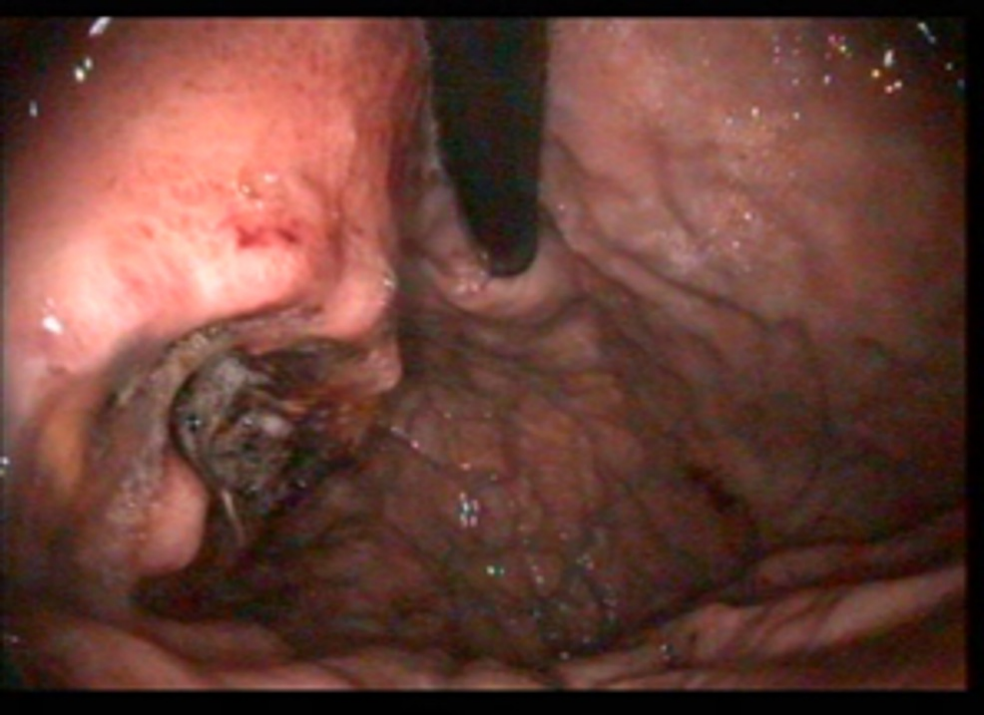
Borrmann Type 2 gastric cancer in the cardia demonstrated by retroflexed gastroscope

A detailed alcohol intake and smoking history was not available in Centre's records. At endoscopy, the antrum and cardia were the common sites affected in 10 (58.8%) and seven (41.2%) cases, respectively. No cancer was noted in the body or fundus of the stomach. All cases of gastric cancer were morphologically advanced at endoscopy. Using the Borrmann classification, type I, type II and type III were seen in eight (47.1%), three (17.6%) and four (23.5%), respectively (Figures 3, 4).

The flat/infiltrating variety (Type IV) was recorded in two (11.8%) cases. Epithelial carcinomas were the most predominant histology with adenocarcinoma recorded in 14 (82.4%) cases and a sole case of Helicobacter pylori detected among all gastric cancer cases. The histology types are as recorded in Table 1.

**Table 1:** Histologic classification of gastric cancers recorded

Histology	Frequency	Percentage
Well/moderately differentiated adenocarcinoma	6	35.3%
Poorly differentiated adenocarcinoma	4	23.5%
Signet ring cell carcinoma	3	17.6%
Squamous cell carcinoma	2	11.8%
Mucinous adenocarcinoma	1	5.9%
Carcinoid tumour	1	5.9%
Total	17	100%

**Figure 5:**
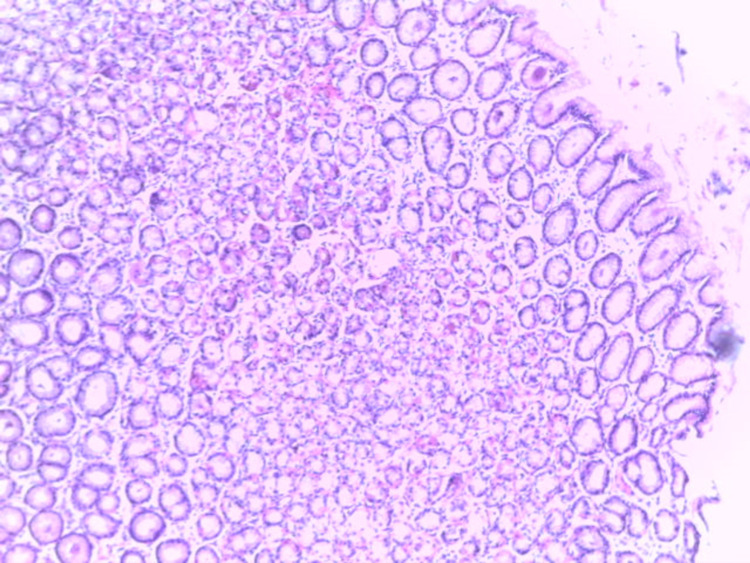
Gastric mucosal biopsy with unremarkable surface epithelial cells and glands adequate in number and distribution (H&E x100) H&E: Haematoxylin and Eosin stain

**Figure 6:**
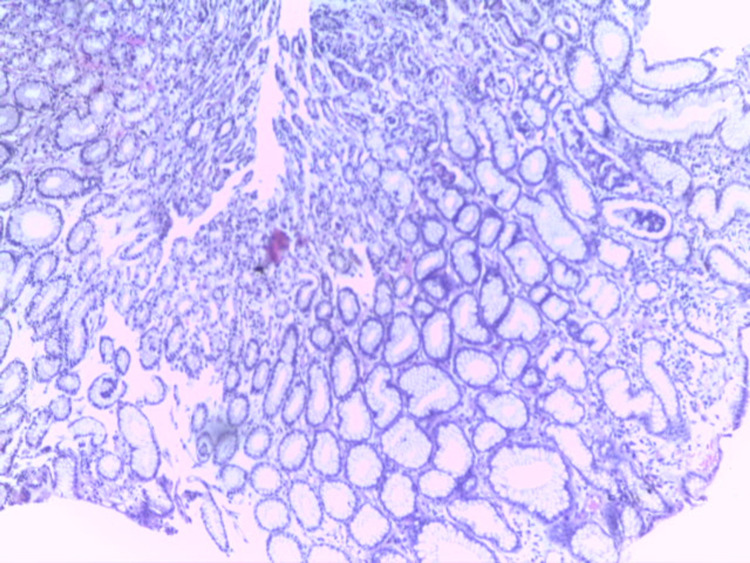
Normal gastric mucosa biopsy (Giemsa stain x200)

**Figure 7:**
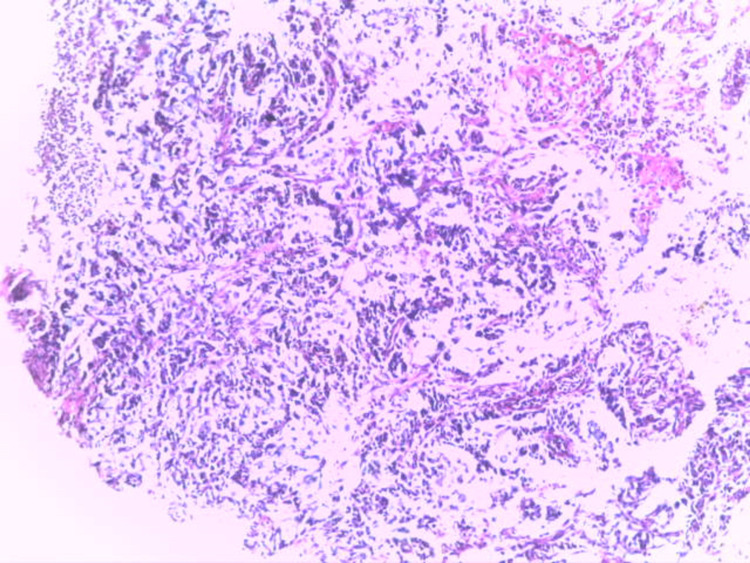
Diffuse type gastric carcinoma showing discohesive sheets of infiltrating malignant epithelial cells with fibrosis of the lamina propria (H&E x100) H&E: Haematoxylin and Eosin stain

**Figure 8:**
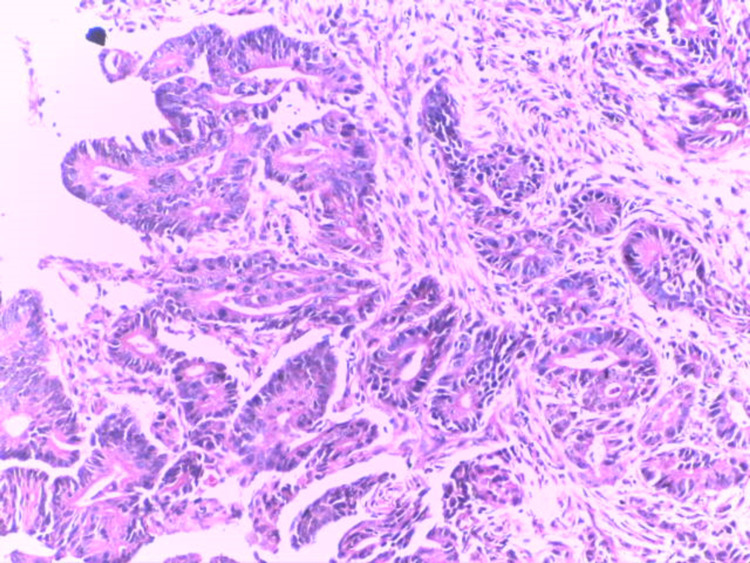
Intestinal type gastric carcinoma showing malignant glands focally fused with marked fibrosis of the lamina propria and infiltration of the muscularis propria (H&E x200) H&E: Haematoxylin and Eosin stain

Using Lauren classification for the cases in Table 1, three of them were reported as diffuse type (Figures 5-8).

Out of the 622 OGD cases, histologic diagnoses of intestinal metaplasia, atrophic gastritis, and Helicobacter pylori were recorded in three (0.5%), six (1.0%), and 142 (22.8%) cases, respectively. No case of mucosa-associated lymphoid tissue (MALT) was recorded.

## Discussion

Gastric cancer is a disease predominantly affecting the middle aged and the elderly with over 70% of new cases and deaths occurring in developing countries [1]. The mean age of patients with gastric cancer recorded in this study was 60.7 years. Several reports from studies in Nigeria have the peak age range of gastric carcinoma in the 6th decade [8,9,20,21]. In East Asia, with the highest reported prevalence of gastric carcinoma, the peak age incidence is the 7th decade for Japan and 8th decade for China and Korea [22-24]. Males were marginally more affected in this study in comparison with major male predominance from other Nigerian studies [8,9,20]. Globally, a male predominance is reported [1]. In view of a similar demographic, socioeconomic and population-based Helicobacter pylori profile in Africa and Far East Asia, the disparity in gastric cancer prevalence is probably diet and gene related.

Epigastric pain and discomfort were the leading complaints from gastric cancer cases in this study with the least duration recorded as one month. Generally, there is lack of early symptoms for gastric cancer with non-specific GI symptom like dyspepsia in 50% of cases [25]. Alarm symptoms include severe weight loss, vomiting and dysphagia and bleeding. In this study, these alarm features constituted more than half of the symptoms recorded. There were no asymptomatic individuals for screening indication in the total population of patients that underwent upper gastrointestinal endoscopy during study period. It is highly likely that premalignant lesions in addition to early gastric cancer could have been detected; hence, an early work-up by upper gastrointestinal endoscopy for especially middle-age patients with dyspepsia and alarm symptoms is essential.

The distal part of the stomach (antrum) was predominantly affected in approximately 60% of gastric cancer cases. This is like studies from Northern and South-western Nigeria [7,8,26]. In developed climes with a high prevalence of gastro-oesophageal reflux disease, there is a higher incidence of proximal stomach and gastroesophageal junction cancers from metaplastic columnar epithelium-lined distal esophageal mucosa secondary to reflux disease [27]. Two cases of upper GI cancers with epicenter in the distal oesophagus but extending into the proximal stomach were excluded and grouped as oesophageal cancers. Based on gross appearance, exophytic growth with defined margins of Borrmann Type I was the predominant morphology in this study. Borrmann’s classification for advanced gastric carcinomas is divided into type I - polypoid growth, type II - fungating growth, type III - ulcerating growth, and type IV - diffusely infiltrating growth [28]. It is important to note that despite macroscopic appearance being informative, the most accurate pre-operative staging information is obtained with endoscopic ultrasonography (EUS) and computer tomography (CT) [29]. In the setting of the study centre, these were not available.

Histologically, there were two cases of the rare histology of squamous cell carcinoma, but adenocarcinoma was most predominant (82.4%). The latter comprised well, moderately, mucinous, and poorly differentiated adenocarcinoma in addition to signet rind carcinoma. The Lauren classification primarily categorizes gastric cancers into intestinal type and diffuse types [28]. The WHO classification was routinely used in histology reports for study patients instead of the Lauren classification. Gastric carcinogenesis is a multistep and multifactorial process with the intestinal type of gastric cancer often related to environmental factors such as Helicobacter pylori infection, diet, and lifestyle, while the diffuse type is more often associated with genetic abnormalities [28]. The intestinal type of gastric cancer is akin to the WHO classification of well differentiated adenocarcinoma-papillary and tubular. Mucinous and poorly cohesive carcinomas (signet cell and poorly differentiated carcinoma) as a group constituted nearly half of the histological varieties of gastric cancer in this study. A large-sized multi-centre study is needed to confirm a possible gene-related pathogenesis in Nigeria.

The limitations encountered in this study include the small sample size and the solely symptomatic population which may challenge the generalization of findings to the entire city population. Also, the white light endoscopy and the non-use of chromoendoscopy may have marred the detection of superficial lesions of early gastric cancer. An electronic chromoendoscopy camera unit was only recently procured in the centre. Another limitation was the non-comprehensive reporting of histology by immunohistochemistry and gene testing for prognostication, up-to-date treatment, and comparison with global cancer library. There is the need for molecular studies on gastric cancer for its pathogenesis in Africa. This study however confirms that chronic dyspeptic symptoms of epigastric pain/discomfort, heart burn, easy satiety, and bloatedness in conjunction with alarm symptoms of weight loss, vomiting and dysphagia are strong indications for the exclusion of gastric cancer by upper GI endoscopy.

## Conclusions

Gastric cancer is uncommon in our environment with a delayed presentation. A predominance of gastric antrum topography and exophytic growth morphology is the pattern in this study population of referred symptomatic patients with a low Helicobacter pylori detection rate. Upper gastrointestinal endoscopy remains very useful in the diagnosis of early-stage gastric cancer. A recommendation is made for an early work-up by upper gastrointestinal endoscopy in middle-age patients with dyspepsia and alarm symptoms of weight loss, vomiting and dysphagia.
